# Modern Subsurface Bacteria in Pristine 2.7 Ga-Old Fossil Stromatolite Drillcore Samples from the Fortescue Group, Western Australia

**DOI:** 10.1371/journal.pone.0005298

**Published:** 2009-04-27

**Authors:** Emmanuelle Gérard, David Moreira, Pascal Philippot, Martin J. Van Kranendonk, Purificación López-García

**Affiliations:** 1 IPGP-IMPMC, Université Denis Diderot et CNRS, Paris, France; 2 Unité d'Ecologie, Systématique et Evolution - CNRS UMR8079, Université Paris-Sud 11, Orsay, France; 3 Geological Survey of Western Australia, East Perth, Western Australia, Australia; University of Wyoming, United States of America

## Abstract

**Background:**

Several abiotic processes leading to the formation of life-like signatures or later contamination with actual biogenic traces can blur the interpretation of the earliest fossil record. In recent years, a large body of evidence showing the occurrence of diverse and active microbial communities in the terrestrial subsurface has accumulated. Considering the time elapsed since Archaean sedimentation, the contribution of subsurface microbial communities postdating the rock formation to the fossil biomarker pool and other biogenic remains in Archaean rocks may be far from negligible.

**Methodology/Principal Findings:**

In order to evaluate the degree of potential contamination of Archean rocks by modern microorganisms, we looked for the presence of living indigenous bacteria in fresh diamond drillcores through 2,724 Myr-old stromatolites (Tumbiana Formation, Fortescue Group, Western Australia) using molecular methods based on the amplification of small subunit ribosomal RNA genes (SSU rDNAs). We analyzed drillcore samples from 4.3 m and 66.2 m depth, showing signs of meteoritic alteration, and also from deeper “fresh” samples showing no apparent evidence for late stage alteration (68 m, 78.8 m, and 99.3 m). We also analyzed control samples from drilling and sawing fluids and a series of laboratory controls to establish a list of potential contaminants introduced during sample manipulation and PCR experiments. We identified in this way the presence of indigenous bacteria belonging to Firmicutes, Actinobacteria, and Alpha-, Beta-, and Gammaproteobacteria in aseptically-sawed inner parts of drillcores down to at least 78.8 m depth.

**Conclusions/Significance:**

The presence of modern bacterial communities in subsurface fossil stromatolite layers opens the possibility that a continuous microbial colonization had existed in the past and contributed to the accumulation of biogenic traces over geological timescales. This finding casts shadow on bulk analyses of early life remains and makes claims for morphological, chemical, isotopic, and biomarker traces syngenetic with the rock unreliable in the absence of detailed contextual analyses at microscale.

## Introduction

In recent years, there has been an intense debate about the authenticity of the oldest claimed traces of life in ancient sedimentary rocks, as most of them, including the accumulation of light isotopes, the presence of organic compounds or that of microfossil-like precipitates, could be simply the product of abiotic processes, particularly those occurring under hydrothermal conditions [1], [2], [3]. The presence of fossil lipids in Archaean rocks cannot be the product of abiotic synthesis and, until recently, was generally accepted as unambiguous evidence for the presence of specific groups of organisms at the time when the sediment deposited. However, ancient life signatures in general might also be the product of later contamination. A recent example highlights this potential problem. In 1999, Brocks and colleagues reported the presence of 2α-methylhopanes and steranes in 2.7 Ga-old rocks from the Pilbara Craton (Australia) that were formerly attributed to 2.7 Gy-old cyanobacterial hopanoids and eukaryotic sterols, respectively [4], [5]. However, recent isotopic δ^13^C values measured at microscale of pyrobitumen and kerogen within the same samples analyzed by Brocks et al. (1999) showed that these are much lighter than values for previously extracted hydrocarbons, suggesting that the latter were later contaminants and reverting the oldest fossil evidence for cyanobacteria and eukaryotes to 2.15 and 1.78–1.68 Ga-old, respectively [6].

There are in principle three potential sources of post-syngenetic contamination by fossil biomarkers and other types of biogenic traces: i) contamination of rock cores during drilling if hydrocarbon-rich oil or kerosene, which also carry isotopic and other life signatures, are used in drilling fluids, ii) migration of fluids containing biogenic signatures to rock layers initially devoid of them, and iii) the accumulation of biomarkers produced in situ by microorganisms inhabiting the rock after its formation. Whereas the first two possibilities have been often considered [7], the latter is generally ignored despite the accumulation of a large body of evidence showing the occurrence of diverse and active microbial communities in the terrestrial subsurface [8], [9], [10], [11], [12]. Here, we contend that, considering the time elapsed since Archaean sedimentation, the contribution of subsurface microbial communities postdating the rock formation to the biogenic signature pool may be far from negligible and that, in some instances, might even preclude hope to discriminate between truly ancient signatures and posterior contaminants.

In order to determine if well-preserved subsurface ancient sedimentary rocks potentially harboring microfossils or other biogenic traces could be considered sterile and exempt of late, post-syngenetic, microbial remains, we investigated by molecular methods based on the amplification SSU rDNAs a suite of pristine diamond drill cores (PDP1 drillcore samples) intersecting Archaean stromatolitic layers that had been collected during the Pilbara Drilling Project from the 2,724 Myr old Tumbiana Formation (Fortescue Group, Hamersley Basin) at Meentheena, Western Australia [13]. The rocks of this and immediately above formations harbor well-preserved fossil stromatolites and have provided a wealth of biogenic signatures, including very negative δ^13^C values presumably linked to Archaean methanotrophic activity [14], filamentous microfossils [15], and the above-mentioned 2α-methylhopanes and steranes that were suggested to derive of 2.7 Gy-old cyanobacterial hopanoids and eukaryotic sterols [4], [5], although not without controversy [6]. Our molecular diversity studies reveal the occurrence of indigenous bacteria in aseptically-sawed inner parts of drillcores of the Tumbiana Formation down to at least 78.8 m depth, which has important implications for the interpretation of the ancient fossil record.

## Results and Discussion

**Figure 1:**
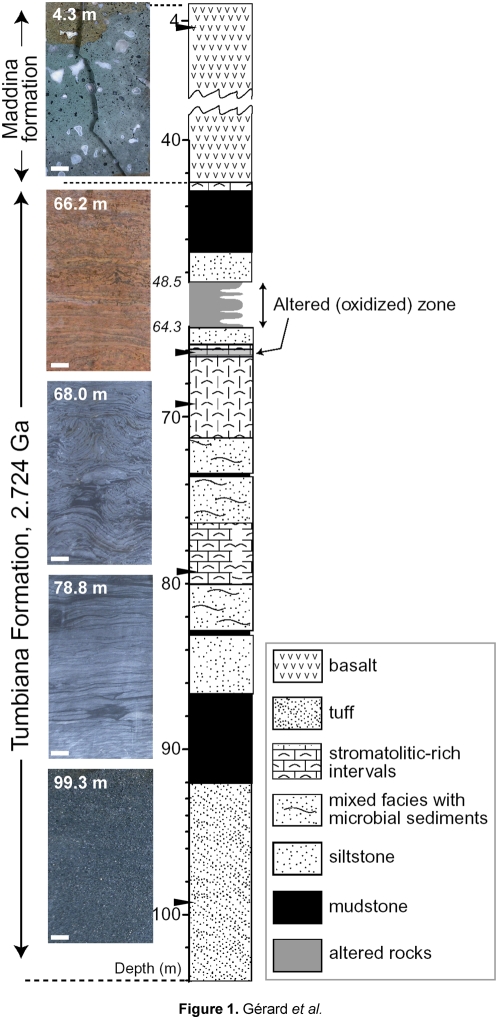
Samples analyzed from the Pilbara Drilling Project drillcore PDP1. Black triangles indicate the regions in the schematic lithological PDP1 log [13] where samples for microbiology analyses were retrieved from. Photographs showing the samples analyzed are shown on the left. The scale bar corresponds to 1 cm.

To look for the putative presence of contemporary microorganisms in 2.7 Ga-old stromatolites, we selected samples from different lithological layers in the field (4.3 m, 66.2 m, 68.0 m, 78.8 m and 99.3 m depth) immediately after the PDP1 core was drilled (Figure 1), which were subsequently treated with maximum precautions to avoid external contamination during sawing and grinding, DNA extraction and subsequent PCR experiments (see Methods). The two upper samples showed evident signs of alteration. The 4.3 m-deep core sample consisted of altered subaerial basalt, and the 66.2 m-deep sample of pervasively weathered reddish stromatolites, which indicated mineral oxidation linked to water circulation, the water table being present approximately until that depth. Deeper samples were apparently unaltered and showed no macroscopic signs of channeled or pervasive fluid infiltration. The 68.0 and 78.8 m-deep samples corresponded to well-preserved dark grey stromatolites consisting of alternated carbonate and mudstone layers. The 99.3 m-deep sample was composed of volcanogenic tuffaceous material [13] (Figure 1).

DNA was extracted from 0.5 g ground core fragments. In the case of stromatolitic layers, (66.2 m and 68.0 m), we performed an additional purification from 10 g of material. To limit the effect of the possible introduction of exogenous microorganisms by the drilling fluid in cores, we sawed them aseptically and retained exclusively the inner part for molecular analyses in the case of the deeper, unaltered samples (68.0 m, 78.8 m and 99.3 m). In addition, to evaluate potential contamination by the drilling fluid, we i) extracted DNA from the drilling fluid used to establish a catalogue of potential drilling-fluid contaminants and ii) analyzed both, inner and outer core parts of the 66.2 m-deep sample. We made no distinction between the inner and outer part for the 4.3 m, which we considered as positive control since, being a shallow altered sample, it might contain more biomass than deeper samples. To establish additional controls of putative laboratory and/or DNA extraction kit contaminants, we subjected blank tubes to the DNA extraction protocols along with drillcore samples and we also extracted DNA from the remaining fluid once the drillcores were sawed. Although the sawing step was a potential source of contamination of core samples, sawing-fluid associated microorganisms may not necessarily be contaminants, but indigenous organisms released from the cores during sawing.

**Figure 2:**
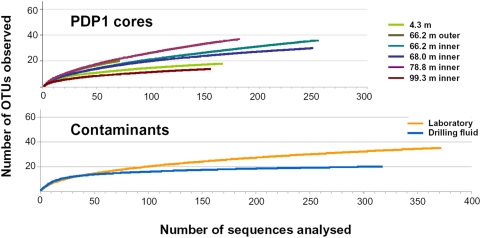
Rarefaction curves of drillcore and control SSU rDNA libraries. The number of operational taxonomic units (OTUs) accumulated can be observed as a function of the number of total sequences obtained. OTUs were defined as clusters of SSU rDNA sequences >97% identical.

**Figure 3:**
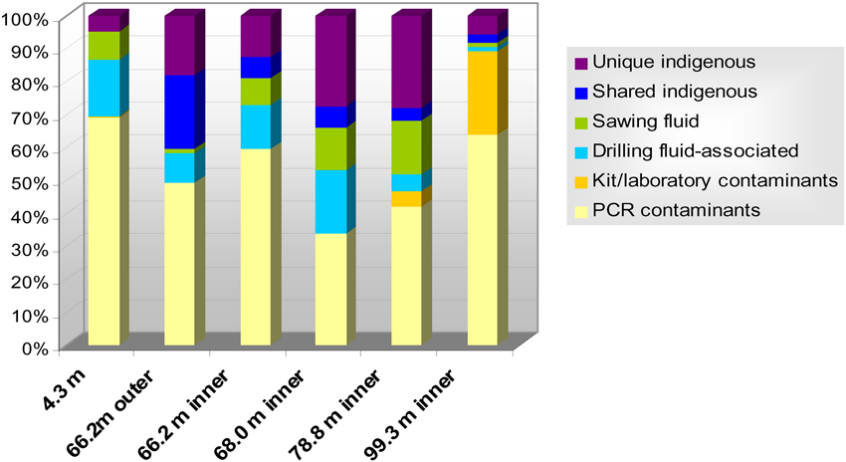
Relative abundance of contaminant and indigenous SSU rDNA sequences in the different PDP1 drillcore sample libraries.

**Figure 4:**
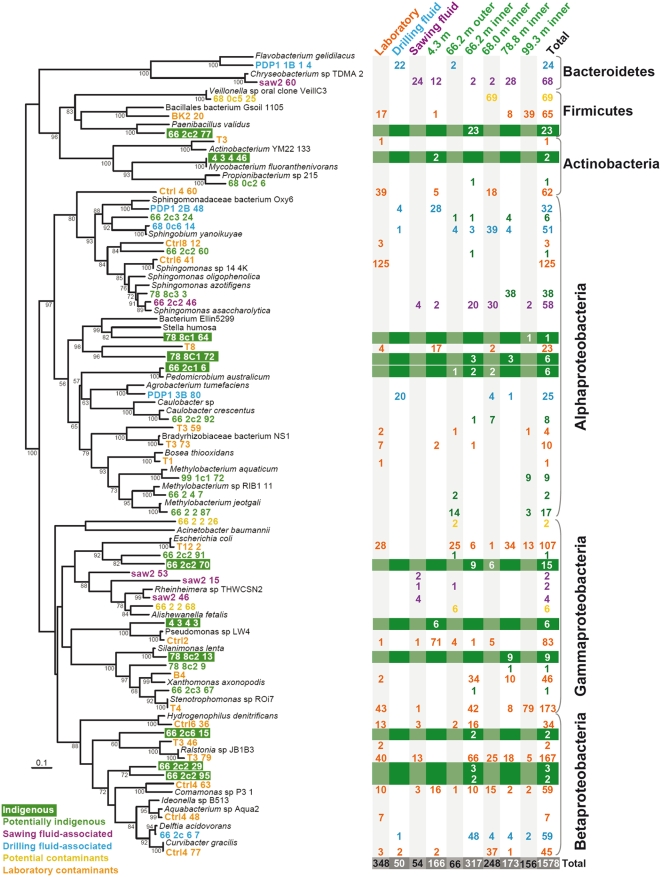
Maximum likelihood phylogenetic tree showing the position of different OTUs identified in PDP1 drillcore and control (laboratory, drilling fluid, sawing fluid) samples. The tree was constructed including the closest possible cultivated members to our sequences. The number of phylotype counts in SSU rDNA libraries from different samples is given on the right. Phylotypes are color-coded attending to their classification along a gradient going from indigenous and potentially indigenous to laboratory contaminants. Clones considered indigenous or potentially indigenous were found exclusively in PDP1 drillcores, having no close relatives (>95% identical) in control contaminant libraries or in the sawing fluid-associated library. Only bootstrap values above 50% are given at nodes.

We amplified SSU rRNA genes from drillcore and drilling and sawing fluid purified DNA. Weak amplification bands were obtained for 4.3 m, 66.2 m and 68.0 m levels, but in too low amount to construct SSU rDNA libraries for molecular diversity purposes. Nested PCR reactions were then carried out to obtain clear amplification bands that served to construct SSU rDNA libraries (Table 1). Being concerned by exogenous contamination problems, we additionally made control SSU rDNA libraries of drilling and sawing fluids as well as laboratory-associated contaminants. The latter comprised 2 libraries of blank control tubes from the DNA extraction kit as well as 6 additional libraries derived from control reactions in nested PCR experiments (controls in direct PCR experiments were all negative) (Table 1). In most cases, we sequenced all the clones that were generated in the different drillcore libraries and, in the case of drilling fluid and laboratory controls, we sequenced clones until a plateau indicating complete coverage of sample biodiversity was reached or nearly so (Figure 2). Based on the list of laboratory and drilling fluid potential contaminants and also in other lists of contaminants in the literature [16], [17], we classified PDP1 phylotypes along a gradient according to their likelihood to be contaminant or truly indigenous (Figures 3 and 4, and Table S1).

**Table 1:** SSU rDNA amplification, libraries generated, and clones yielded and/or analyzed in PDP1 drillcores of different depths.

	Direct PCR			Nested PCR			Number of OTUs
Samples	PCR product	No. libraries produced	No. clones obtained/analyzed	PCR product	No. libraries produced	No. clones obtained/analyzed	
**Drill core samples**							
4.3 m	+/−	2	17	+	3	63	2
66.2 m outer	+/−	1	3	+	4	147	7
66.2 m inner	+/−	1	8	+	6	308	12
68.0 m inner	+/−	1	4	+	5	244	3
78.8 m inner	−	−	−	+	4	173	5
99.3 m inner	−	−	−	+	5	155	3
**Control samples**							
Sawing fluid	+/−	−	−	+	3	56	5
Drilling fluid	+	7	317	nd	nd	nd	20
Laboratory*	−	−	−	+	8	367	19

* (PCR+DNA extraction kit) associated contaminants

**nd** not done

OTU, operational taxonomic unit.

Laboratory contaminants coming from the surrounding environment, PCR reagents or the DNA extraction kit accounted for a relative high proportion of sequences in SSU rDNA libraries (Figure 3) belonging mostly to Gram positive bacteria and various subdivisions of the Proteobacteria (Figure 4). Most of them affiliated to the genera *Sphingomonas, Stenotrophomonas, Ralstonia,* to a divergent alphaproteobacterium (clone 4_60) and to *Shigella/Escherichia,* the latter likely deriving from trace amounts of *E. coli* DNA in recombinant Taq polymerase aliquots as it was associated to PCR controls, and they comprised 36%, 16%, 11%, 11%, and 8% of the total contaminant clones, respectively. Although not found in laboratory control libraries, we also discarded clones that shared more than 95% sequence identity with animal or human-related bacteria as potential contaminants (Figure 4 and Table S1). Contaminants introduced by the drilling fluid comprised moderate proportions that were observed in all the drillcore samples down to 99.3 m depth. The proportion of drilling fluid-associated phylotypes was not significantly higher in the outer part of the 66.2 m-deep core compared to its inner part and was comparable to that of the 4.3 and 68.0 m-deep layers (Figure 3). This suggests that, the porosity of the different samples being very low [18], contamination by the drilling fluid was not extensive in the rock and that contamination of both, inner and outer part of cores, occurred through microfractures. Phylotypes identified in the drilling fluid affiliated to the Sphingomonadaceae family (48% of the total drilling fluid clones) or were related to the genera *Delftia* (24%), *Agrobacterium* (14%) and, distantly, *Flavobacterium* (14%). Although we considered them as contaminants introduced by the drilling fluid, microorganisms belonging to the Sphingomonadaceae family or to *Delftia* species are ubiquitous, being frequently isolated from aquifers [19], [20], and the possibility that they might be found not only in the water used for drilling but also in the water circulating in the subsurface cannot be completely excluded. This caution might apply also to some phylotypes that we have considered here as laboratory contaminants. For instance, we identified a phylotype closely related to *Hydrogenophilus* spp. (Figure 4, Table S1), moderately thermophilic hydrogen and sulfur-compound oxidizers [21] associated to subsurface aquifers, oil wells and even, perhaps, the lake Vostok [22]. Although we consider it here as a contaminant, *Hydrogenophilus* relatives are also present in the subsurface aquifer of the Great Australian Artesian Basin [23], and might therefore be actually present in PDP1 samples. Phylotypes detected in the sawing-associated fluid were not considered as indigenous subsurface microbes and were classified apart, although the possibility that they come from the core samples themselves cannot be discarded.

Phylotypes found in drillcores that were not detected in control libraries but that were found only once or belonged to ubiquitous lineages having relatives identified also in contaminant libraries from literature data were considered only as potentially indigenous. This was the case of several alphaproteobacterial sequences shared by samples coming from 66.2 m to 78.8 m depth that formed 3 OTUs affiliated to the genus *Sphingomonas* or *Sphingobium*. Species of these genera are ubiquitous aerobic, chemoheterotrophic bacteria having been isolated from various environments including the deep subsurface [19], [20], [24], [25] but also clean rooms where space craft are assembled [17]. Similarly, various sequences forming three OTUs affiliated to the genus *Methylobacterium* were considered only as potentially indigenous (Figure 2 and Table S1). Although members of this genus have been detected as contaminant in some libraries in the literature [26], their distribution is widespread in many different environments, and moderate thermophilic *Methylobacterium*-like alphaproteobacteria have been isolated from deep aquifers in Australia [27].

Finally, we classified as truly indigenous to the Tumbiana subsurface, sequences found in general at several core depths or retrieved from independent clone libraries. Several of these phylotypes were more or less distantly related to Alpha-, Gamma- and Betaproteobacteria, Firmicutes and Actinobacteria sequences identified in soil but also in hot springs or subsurface sediments (Figure 4 and Table S1). One Firmicutes phylotype was distantly related to *Bacillus* and *Paenibacillus* sequences retrieved from subsurface sediments [28]. Iron and manganese reducing *Bacillus* species have been isolated from deep aquifers in Australia [29]. We also detected phylotypes closely related to the alphaproteobacterium *Pedomicrobium australicum* in samples 66.2 and 68.0 m. *Pedomicrobium* species are manganese and sometimes iron oxidizing bacteria able to colonize a wide variety of environments including desert rocks [30], [31]. Some of them can also metabolize methanesulfonic acid, a capability also shared by *Methylobacterium* species [32], and sulfur-containing aromatic compounds [33], playing also a role in the sulfur cycle. Since iron, manganese and sulfur compounds are abundantly available in these Archaean rocks, they might be used as electron donors by a variety of subsurface microorganisms. The other two indigenous alphaproteobacterial phylotypes identified were only very distantly related to any described species or environmental sequences, and might thus represent new lineages of subsurface-associated microorganisms. This was also the case of Betaproteobacteria indigenous phylotypes, which were only distantly related to environmental sequences retrieved from geothermal areas or compost. Among the Gammaproteobacteria, we identified three phylotypes indigenous to the drillcores. One of them was closely related to *Silanimonas lenta*, isolated from a hot spring, and to a Xanthomonadaceae sequence (Acc.No. DQ230964) retrieved from subsurface water in Kalahari, South Africa.

The fact that we amplified indigenous bacterial DNA in our samples did not prove that these bacteria are actually alive and active. They might be dormant or the detected DNA could correspond to the remains of dead bacterial cells. However, DNA is a relatively unstable molecule that may be rapidly degraded after the death of the microorganisms, first by endogenous nucleases and, then, by chemical processes like hydrolysis or oxidation [34]. In certain conditions of desiccation or high salinity, DNA may be more stable and several studies have claimed that bacterial DNA could survive more than 10 millions years [35], [36], [37], [38] but, in addition of being highly controversial claims [39], [40], a few million years is a short time in the geological record, especially when 2,724 Myr old Archaean rocks are being considered. In addition, fossil DNA is characterized by being split in small fragments by the above-cited hydrolytic processes. Since we amplified 1465-bp long DNA sequences, it is highly improbable to obtain such long amplification from fossil DNA older than few thousand years [41]. Furthermore, several of our indigenous or potential indigenous candidate SSU rDNAs share up to 99% identity with those of other modern bacteria. If these sequences were truly derived from the amplification of Archaean bacteria, they should have diverged more that 1% at this locus since the deposition of these rocks some 2,724 Myr ago. Such low evolutionary rate at this locus is incompatible with current knowledge of gene evolution. We thus conclude that the DNA we detected in our core samples comes from modern bacteria that are alive or were still alive few thousand years ago.

How many contemporary cells might be present in Hamersley subsurface fossil stromatolites? This is very difficult to estimate due to a likely low cell density combined with the difficulties associated to the study of subsurface rock-associated bacteria. By direct PCR, we only detected weak amplification bands in some samples, in agreement with the presence of low biomass even in altered samples. Direct counting of bacterial cells using epifluorescence microscopy and DNA labeling with fluorochromes such as DAPI or Syto9 was not possible. Carbonate-rich mineral samples exhibit high autofluorescence and have affinity for usual DNA stains used to detect microbial cells, making it very difficult to distinguish bacterial cells from mineral particles in carbonate rocks, particularly in samples where the biomass is extremely low, as was likely the case. The combination of carbonate autofluoresce and low cell density prevents also the use of metabolic dyes that target intact cell membranes such as the LIVE/DEAD® Viability kit (Molecular Probes™) or that of fluorescent in situ hybridization (FISH) experiments using specific probes, making it extremely difficult to distinguish between artefactual binding from true cell labeling. This kind of detection is further impeded if we consider that cells present in the rock may be dormant or have extremely low metabolic rates. This would imply that metabolic staining may be below detection limits. Similarly, cells with low or no detectable metabolic activity may have too few ribosomes for FISH probes to produce a detectable signal. Therefore, we cannot provide precise estimates of cell numbers in our samples but the weak amplification signal obtained in most PCR experiments suggests that cell density is probably very low. For comparison, microbial cell densities of ca. 10^2^–10^4^ cells/g rock have been estimated in ultradeep mines [16] and other continental drilling samples [12], [42].

Our results strongly suggest that contemporary bacteria inhabit what are generally considered exceptionally well-preserved subsurface Archaean fossil stromatolites of the Hamersley Basin, Western Australia. They are possibly in very low numbers, their distribution confined to microfractures where water may circulate (perhaps only intermittently), and their metabolic activities might be extremely low. However, upon geological timescales spanning 2.7 Gy, even such low cell numbers must have contributed significantly to the pool of biogenic signatures associated to these rocks, including microfossils, biological isotopic fractionation and lipid biomarkers. Although our results do not necessarily invalidate previous analyses, they cautiously question the interpretation of ancient biomarkers or other life traces associated to old rocks, even pristine, as syngenetic biogenic remains when bulk analyses are carried out. In this context, the development of high-resolution techniques that allow the identification of biogenic signatures in microscale-preserved environments is particularly promising. For example, high resolution spectroscopy and microscopy analyses revealed the presence of aragonite nanocrystals closely associated with organic nanoglobules, supporting a microbial role for stromatolite formation within well-preserved 68.2 m-deep stromatolitic layers in the same PDP1 drillcore that we have studied here [18], and measurements of carbon isotope deviations at microscale have allowed the discrimination between syngenetic organic matter and later biomarker contaminants in Archaean sedimentary rocks, eliminating false positives and paving the way for more accurate datation based on fossil biomarkers in the future [6].

## Methods

### Sample collection and preparation

The samples analyzed in this study were obtained in August 17th and 18th 2004 from one diamond drillhole section (PDP1) that intersected one stromatolitic horizon through the c. 2.72 Ga Tumbiana Formation (Fortescue Group, Mount Bruce Supergroup, Hamersley Basin). Sampling was made possible within the framework of the Pilbara Drilling Project between the Institut de Physique du Globe de Paris and the Geological Survey of Western Australia. The detailed geological log of the diamond drillhole section has been reported [13]. Briefly, PDP1 intersected subaerial basalts of the Maddina Formation, stromatolitic carbonates and interbedded black shale of the Meentheena Member of the Tumbiana Formation, and underlying volcaniclastic sandstones. Stromatolites were identified by domical shapes and wrinkly laminations, including black organic-rich laminae. Local water from the Nullagine river was used for drilling. PDP1 was drilled with NQ2 core (47.6 mm diameter) from the surface to a depth of 104 m (Figure 1). Drillcores were extracted with maximum precautions to avoid external environmental contamination. All manipulation with bare hands by the operators of the Mount Magnet Drilling Company was prevented and cores were directly deposited in clean steel core trays. Digital photographs and lithological logs of the core were made in the field. Core sections selected for microbiological analyses were immediately separated using clean laboratory gloves and preserved in sterile plastic bags from external contact during transportation of core trays to the Geological Survey in Perth. Selected core fragments were sawed to extract the inner core (ca. 2 cm×2 cm×10 cm) for DNA extraction and analysis of microbial diversity. Prior to use the saw was treated extensively with 5% sodium hypochlorite for several hours and then rinsed with sterile mineral water. The saw reservoir was then filled with sterile mineral water and mixed with sodium hypochlorite to reach a diluted solution (ca. 0.1%). Core surfaces were cleaned with ethanol prior to sawing. During sawing the cores were hold with clean laboratory plastic gloves avoiding touching the inner core, which was finally collected using heat-sterilized forceps. Inner core surfaces were then pressed against a sterile gaze soaked with 5% sodium hypochlorite and the inner cores introduced in 50-ml Falcon tubes and fixed with 96% ethanol from a newly opened bottle. Treatment with 5% sodium hypochlorite was found to be the most efficient protocol for decontamination of external contaminating microbes and nucleic acids while maintaining those of internal microbes in ice cores [43]. Outer core sections were kept dry in sealed sterile plastic bags. After their transportation to the microbiology laboratory at the University of Paris-Sud, Orsay, France, all samples were stored at −20°C until use.

In addition, samples of the drilling fluid and sawing water used were also collected and fixed in situ to establish control SSU rRNA gene libraries of potential microbial contaminants introduced during the drilling process. The drilling fluid consisted of a mixture of river water with silicon oil. No drilling mud or diesel was put down the hole during drilling to avoid undue contamination by foreign organic material. The drilling fluid, which was at a pH of 7.0, was collected directly from the tank feeding the diamond drillcore during the drilling operation at Pilbara. 50 ml were filtered through a TMMP Millipore 5 µm-pore size filter and then through a GTTP Millipore 0.2 µm-pore size filter in a field laboratory set for this purpose. All the materials used were sterile. Upon filtration, the filter was fixed in ethanol for preservation and transport until the microbiology laboratory at Orsay, France. Filters were then stored at −20°C until use. Similarly, ca. 25 ml of the water used for core sawing containing a high density of rock particles in suspension was collected at the Geological Survey laboratory in Perth immediately after sawing, fixed with two volumes of 100% ethanol for transportation to France and, upon arrival, stored at −20°C.

### DNA extraction, PCR amplification, and cloning

All sample manipulations carried out at the University of Paris-Sud, including DNA extraction and derived DNA-based protocols were done in a Biocap™ RNA/DNA hood (Captair, Erlab, France), a vertical laminar flow chamber specifically designed for the manipulation of samples with high contamination risk, equipped with a HEPA filter that guarantees 99,999% filtration efficacy for particles larger than 0,3 µm in diameter. Prior to use the chamber was UV-sterilized. All laboratory materials and solutions used were sterile (most often sterilized twice). Aerosol resistant pipette tips were always used to reduce the likelihood of external contamination. We selected inner core samples from 4.3, 66.2, 68.8, 78.8 and 99.3 m depth representing characteristic layers in the log (Figure 1). In addition, the outer core fragments of the 66.2 m-deep sample, corresponding to altered stromatolitic layers, was also analyzed (Table 1). Initially, inner cores were fragmented into pieces and ground finely in a sterile agatha mortar. 0.5 g of drillcore powder from each level was used for molecular diversity studies. Subsequently, due to the low yield of PCR and cloning steps for deep cores, we additionally used 10 g of powder from deep stromatolitic layers 66.2 m (altered) and 68.0 m (non-altered) inner core samples. 1.4 g of wet decanted powder from the sawing associated-fluid was used for DNA extraction, which was then mixed with the DNA extracted from a GTTP 0.2 µm pore-size filter where the potential microbial biomass from the remaining sawing water fixed in ethanol was filtered through. DNA of the different samples, including 0.2 µm pore-size filters used to collect the biomass from drilling and sawing fluids cut into pieces, was extracted using the PowerSoil DNA isolation kit (MoBio Laboratories) with minor modifications. DNA was resuspended in 50 µl of sterile 10 mM Tris/HCl, pH 8.5, and conserved at −20°C. As a laboratory control of potential contaminants, for each different DNA extraction done, a blank tube was subjected in parallel to the same extraction protocol applied to drillcore samples in order to construct control clone libraries from kit material and/or other laboratory-associated contaminants that might be introduced in stromatolite samples during DNA purification. Two laboratory control samples were produced in this way and subjected to PCR amplification protocols simultaneously to drillcore DNA samples.

Bacterial SSU rRNA genes were amplified by PCR using different combinations of the bacterial-specific primers 27F (5′-AGAGTTTGATCCTGGCTCAG) or 63F (5′-CAGGCCTAACACATGCAAGTC) and the prokaryote-specific reverse primers 1492R (5′-GGTTACCTTGTTACGACTT) or 1397R (5′-GGGCGGWGTGTACAAGGC). 5 µl of DNA purified as described above were used in 25–30 µl-volume PCR reactions using either GoTaq polymerase (Promega, France) or the hot-start Platinum Taq (Invitrogen), always opening new aliquots for each set of drillcore SSU rDNA amplification reactions. These were performed under the following conditions: 30 cycles (denaturation at 94°C for 15 s, annealing at 55°C for 30 s, extension at 72°C for 2 min) preceded by 2 min denaturation at 94°C, and followed by 7 min extension at 72°C. Direct PCR reactions were carried out with different primer combinations and, when applicable (Table 1), 1 µl of the first PCR reaction carried out with the most external primers (27F and 1492R) was then used in nested PCR reaction using the same conditions with primers 63F and 1387R. Negative controls were set for all PCR reactions, all of which were negative in direct PCR reactions. Several negative controls in nested PCR amplification experiments yielded amplicons, which were then cloned to contribute establishing a catalogue of laboratory and/or PCR reagent contaminants. In total, eight libraries of PCR-associated laboratory contaminants were constructed. From them, a relative high proportion of these clones yielded sequences closely related to *Escherichia coli*, suggesting that traces of DNA may persist in Taq polymerase commercial aliquots. Cloning was done using the Topo TA Cloning system (Invitrogen) following the instructions provided by the manufacturers. After plating, positive transformants were screened by PCR amplification of inserts using flanking vector primers and PCR products partially sequenced using either 1387R or 1492R (Cogenics, France). Table 1 is a summary of the PCR amplification results, SSU rDNA libraries constructed and the number of clones obtained and/or analyzed per library corresponding to the different drillcore samples, the drilling and sawing fluids as well as laboratory (PCR reaction+DNA extraction kit) associated contaminants. Sequence data have been deposited in GenBank (http://www.ncbi.nlm.nih.gov/) with accession numbers FJ854696-FJ854749.

### Phylogenetic and statistical analyses

Only high-quality partial sequences (700–800 bp) were retained for subsequent analyses. We discarded sequences of poor quality or potential chimeras. This yielded a total of 1578 sequences (Figure 2) that were used for phylogenetic and statistical analyses. Partial sequences were compared to those in databases by BLAST [44]. Multiple alignments were carried out using MUSCLE [45] and manually edited using the program ED from the MUST package [46]. Preliminary distance (neighbor-joining) trees allowed the identification of groups of highly similar sequences (>97% identity) or phylotypes. One representative clone from each phylotype was fully sequenced. Complete sequences were used to reconstruct a maximum likelihood phylogenetic tree with TREEFINDER [47], applying a general time reversible model of sequence evolution (GTR), and taking among-site rate variation into account by using an six-category discrete approximation of a Γ distribution (Figure 2). ML bootstrap proportions were inferred using 1000 replicates. Rarefaction curves were estimated out of the distance matrix from the different sequence alignments using the program DOTUR and the conventional species cut-off limit (members of a same OTU have >97% SSU rDNA sequence identity) [48].

## Supporting Information

Table S1 (0.07 MB PDF)
