# Supplementary material for: Modern Subsurface Bacteria in Pristine 2.7 Ga-Old Fossil Stromatolite Drillcore Samples from the Fortescue Group, Western Australia
Source: PLoS One. 2009 Apr 27;4(4):e5298. doi: 10.1371/journal.pone.0005298 (PMC2671143; doi:10.1371/journal.pone.0005298)
Supplement: Table S1 — (0.07 MB PDF) [file pone.0005298.s001.pdf]

Supplementary Table S1.

Affinities of representative clones of the OTUs identified in the PDP1 drilled samples and the potential contamination sources analysed in this study.

Clone name	Closest BLAST hit [Accession number]	% identity	Type of environment of origin	Phylogenetic ascription
4 3 4 3	*Pseudomonas extremeaustralis* CT14-3 [AJ583501]	96	Antarctic, high stress resistant strain	Gammaproteobacteria
4 3 4 46	Bacterium 1a-G2 [AB354138]	99	manganese nodules in rice field subsoils	Actinobacteria
66 2C1 6	*Pedomicrobium australicum* [X97693]	99		Alphaproteobacteria
66 2C2 29	Uncultured bacterium EU340201 [EU340201]	95	associated to aquatic macrophyte in geothermally active area	Betaproteobacteria
66 2C2 70	Uncultured bacterium FFCH5949 [EU134717]	92	soil from undisturbed mixed grass prairie	Gammarpoteobacteria
66 2c2 77	Firmicutes *Bacillus sp. HSCC* [AB045095]	94		Firmicutes
66 2C2 95	Uncultured bacterium clone SMB80 [AM183046]	87	compost	Betaproteobacteria
66 2C6 15	Uncultured Betaproteobacterium CrystalBog2G3 [AY792245]	96	humic lake	Betaproteobacteria
78 8C1 64	Uncultured bacterium FFCH1465 [EU133427]	93	soil from undisturbed mixed grass prairie	Alphaproteobacteria
78 8c1 72	Uncultured bacterium FFCH1465 [EU133427]	93	soil from undisturbed mixed grass prairie	Alphaproteobacteria
78 8C 2 13	Silanimonas lenta [AY557615]	97	hot spring	Gammaproteobacteria
66 2 4 7	Phenanthrene-degrading bacterium M10 [AY177363]	99	soil column system	Alphaproteobacteria
66 2 2 87	Uncultured bacterium YCB61 [EF205468]	99	geothermal spring mat	Alphaproteobacteria
66 2C2 60	Sphingomonas oligophenolica [AB365794]	94	Granite soil of Korean ginseng rhizosphere	Alphaproteobacteria
66 2C2 91	*Photorhabdus luminescens* [AY594267]	90		Gammaproteobacteria
66.2c2 92	Caulobacter vibrioides [EU730905]	99	water purification system	Alphaproteobacteria
66.2C3 24	Sphingobium yanoikuyae [EU307932]	98	soil	Alphaproteobacteria
66 2c3 67	Stenotrophomonas strain CTF24 [EU294137]	96	saline agricultural farmland soil	Gammaproteobacteria
68 0C2 6	Uncultured Hydrocarbon seep bacterium BPC009 [AF154099]	93	Hydrocarbon seep	Actinobacteria
78 8C2 9	Pseudoxanthomonas taiwanensis [AM932276]	95	mature mushroom compost	Gammaproteobacteria
78 8c3 3	Sphingomonas azotifigens [AB217472]	97	roots of Oryza sativa	Alphaproteobacteria
99 3C1 72	Uncultured Methylobacterium sp. clone YJQ-19 [AY569294]	98	pink mat from hot spring	Alphaproteobacteria
saw2 60	Uncultured bacterium clone LR A2-10 [DQ988291]	99	biofilm system for coking wastewater treatment	Bacteroidetes
66 2C2 46	*Sphingomonas asaccharolytica* [Y09639]	97		Alphaproteobacteria
saw2 53	Gammaproteobacterium GPTSA100-22 [DQ914845]	95	warm spring	Gammaproteobacteria
saw2 15	Uncultured Ralstonia sp. 1P-1-B16 [EU704724]	93	Kennedy Space Center clean room floor	Gammaproteobacteria
saw2 46	Gammaproteobacterium GPTSA100-22 [DQ914845]	98	warm spring	Gammaproteobacteria
PDP1 1B 14	Flavobacterium cheniae [EF407880]	96	China: sediment of Guanting Reservoir, Beijing	Bacteroidetes
PDP1 3B 80	Rhizobium sp. W3 [EU781656]	99	ground water	Alphaproteobacteria
PDP1 2B 48	Uncultured bacterium clone KSC2-93 [DQ532296]	98	Kennedy space center	Alphaproteobacteria
66 2C6 7	Uncultured bacterium nbt120c08 [EU540588]	99	skin	Betaproteobacteria
68 0C 6 14	Sphingobium yanoikuyae [EU307932]	99	soil	Alphaproteobacteria
66 2 2 68	Uncultured bacterium clone PB2_aai21e08 [EU460485]	95	polar bear feces	Gammaproteobacteria
68 0C5 25	Uncultured bacterium clone P2D1-689 [EF510438]	99	Homo sapiens	Firmicutes
66 2 2 26	Uncultured Neisseriaceae bacterium clone LR2-63 [DQ847448	99	human skin	Gammaproteobacteria
BK2 20	Uncultured Firmicutes MoB-G6-114 [EF016847]	99	method control sample in Atacama arid desert study	Firmicutes
T3	Uncultured bacterium 1013-28-CG55 [AY532590]	97	uranium-contaminated aquifer	Actinobacteria
T3 73	[U670061]	99	3-µm prefiltered seawater, 120 m depth above the Kerguelen plateau, Southern Ocea	Alphaproteobacteria
ctrl8-12	Sphingomonas sp. 14_4K [EF540484]	96	solid waste from oil-shale chemical industry	Alphaproteobacteria
Ctrl6-41	Sphingomonas sp. 14_4K [EF540484]	99	solid waste from oil-shale chemical industry	Alphaproteobacteria
T8	Uncultured bacterium clone 015C-B05 [AY662021]	92	groundwater contaminated with high levels of nitric acid-bearing uranium waste	Alphaproteobacteria
T1	Uncultured bacterium clone B3NR69D20 [AY957935]	99	drinking water biofilm	Alphaproteobacteria
T4	Stenophotromonas maltophilia [EU294137]	99	saline agricultural farmland soil	Gammaproteobacteria
B4	Uncultured bacterium clone 21f06 [EF515229]	99	upflow microbial fuel cell anode	Gammaproteobacteria
ctrl 2	Uncultured bacterium clone nbt188c03 [EU535023]	99	mouse skin	Gammaproteobacteria
ctrl 6 36	Uncultured Betaproteobacterium [EF188435]	99	Altamira cave	Betaproteobacteria
ctrl4 60	Uncultured bacterium clone EV818CFSSAHH36 [DQ336987]	91	subsurface water	Alphaproteobacteria
ctrl4 63	*Pseudomonas lanceolata* [AB021390]	95		Betaproteobacteria
ctrl4 77	Uncultured bacterium clone HDBW-WB16 [AB237679]	99	deep subsurface water from sedimentary rock milieu	Betaproteobacteria
ctrl 4 48	Aquabacterium sp. Aqua2 [AF089858]	99	drinking water biofilm	Betaproteobacteria
T3 79	*Ralstonia pickettii* [CP001069]	99		Betaproteobacteria
T3 59	Uncultured bacterium clone B4M56D3 [AY957944]	99	drinking water biofilm	Alphaproteobacteria
coli103 2 / T12 2	Shigella sonnei [EU723822]	99	hemp retting water	Gammaproteobacteria

Indigenous
Potentially indigenous
Sawing fluid associated
Drilling fluid associated
Potentially contaminant
Laboratory contaminant
